# 642. Real-time Data Capture

**DOI:** 10.1093/jbcr/irag033.479

**Published:** 2026-04-06

**Authors:** Antonio Kalk, Nicole M Kopari

**Affiliations:** Leon S. Peters Burn Center, Fresno, California; Leon S. Peters Burn Center, Fresno, California

## Abstract

**Introduction:**

American Burn Association Verification requires data input into the Burn Care Quality Platform (BCQP) registry. Burn Centers employ Registrars to perform chart reviews and pull data points for entry. As our Registrar is a non-clinical position, it emphasizes the importance of appropriate documentation within the electronic medical record (EMR) which is often unreliable or incomplete. To overcome this reliance on retrospective chart review, we developed a novel way of gathering data in real-time by having the Registrar attend daily rounds, transforming a non-clinical role into an active data collection and quality assurance position.

**Methods:**

The Registrar position for our Verified Burn Center job description requires 1-year of computer and medical experience and successful completion of an anatomy course. This does not correlate into clinical experience. Burn patients often experience prolonged hospitalizations resulting in lengthy chart reviews for our Registrar. We implemented a “Plan of the Day” sheet, which burn team members use on rounds to update the nursing staff of any changes regarding patient care. We added a section highlighting liberation rounds and BCQP data points to ensure documentation and discussion in real-time.

**Results:**

Our Registrar attends rounds Monday–Friday. The “Plan of the Day” sheets help prompt clinicians to discuss BCQP data points as part of the daily rounds discussions. The Registrar’s presence ensures immediate clarification of documentation gaps and fosters a continuous feedback loop between clinical and data-focused team members. This allows real-time collection of data and encourages clinicians to update the problem list in the EMR. BCQP data is then discussed monthly at the Burn QI/PI meeting. If trends are noted, action plans are created and loop closure is documented to ensure satisfactory patient outcomes. This process was highlighted during our most recent verification. The surveyors noted that having the Registrar play such an integral part of the team should become standard of care or best practice.

**Conclusions:**

Registrars not only engages with the burn team members but becomes an integral part of the burn team by tracking data points and outcomes in real-time. Creating a document listing the BCQP data points decreased the need for lengthy chart reviews allowing the Registrar to focus on other quality initiatives and process improvements. This enhanced collaboration and immediate feedback mechanism improved both data quality and team cohesion.

**Applicability of Research to Practice:**

This model provides a high-impact workflow change that directly addresses common challenges in accreditation and quality management by establishing a new best practice for data integrity. Its success is highly scalable and immediately applicable for all ABA Verified Burn Centers seeking to optimize BCQP data quality and improve patient outcomes through proactive, embedded quality assurance.

**Funding for the study:**

N/A.

